# Using Data Assimilation for Quantitative Electroencephalography Analysis

**DOI:** 10.3390/brainsci10110853

**Published:** 2020-11-12

**Authors:** Lizbeth Peralta-Malváez, Rocio Salazar-Varas, Gibran Etcheverry, David Gutiérrez

**Affiliations:** 1 Department of Computing, Electronics and Mechatronics, Universidad de las Américas Puebla, San Andrés Cholula, Puebla 72810, Mexico; rocio.salazarv@udlap.mx (R.S.-V.); gibran.etcheverry@udlap.mx (G.E.); 2 Center for Research and Advanced Studies (Cinvestav), Monterrey’s Unit Apodaca, Nuevo León 66600, Mexico; dgtz@ieee.org

**Keywords:** data assimilation, quantitative electroencephalography, Ensemble Kalman filter, neurocognitive processes

## Abstract

We propose a method based on the ensemble Kalman filter (EnKF) together with quantitative electroencephalogram (QEEG) coherence and power spectrum analysis for evaluating changes in brain activity associated with cognitive processes. Such analysis framework has been widely used in the context of data assimilation (DA) in areas such as geosciences, meteorology, and aerospace. However, the use of this approach is less common in neurosciences. In our case, EnKF highlights the spectral contribution of brain signals that are more likely (according to their coherence analysis) to be related to the cognitive process of interest. The power enhancement, due to the cognitive activity, is later validated in the power spectrum analysis by comparing through statistical tests relevant frequency content in two datasets in which assessing the development of cognitive abilities is of interest: the process of getting concentrated and of learning a new skill. Our results show that our DA-based methodology can highlight important frequency characteristics of the electroencephalogram (EEG) data that have been related to different cognitive processes. Hence, our proposal has the potential to understand of neurocognitive phenomena that is tracked through QEEG.

## 1. Introduction

Data assimilation (DA) is a technique that combines numerical data modeling and observations to obtain an accurate representation of the phenomenon of interest [1]. Typically, model parameters and conditions are used for representing an event. Instead, in the context of DA framework, these are used for the prediction of the next state. Such a forecast is updated with the DA and new observations for analysis purposes. Next, the model is restarted with this analysis and a new prediction is computed. This cycle is repeated until all the observations have been assimilated. Some of the methods used are optimal interpolation, 3D or 4D variational method, and Kalman filter (KF), along with its variants like extended KF (EKF), unscented KF (UKF), and ensemble KF (EnKF) [2]. This last one is the method of interest for this research.

DA has been widely used in areas like aerospace, navigation systems, oceanography, meteorology, and geosciences [3]. Some articles declare that the researchers are recently using the DA methods to analyze the brain activity [4,5,6]. Nevertheless, in the late 1970s, KF was used for studying the power spectrum in the electroencephalogram (EEG) signals [7]. It is true that if we compare the use of DA methods in the neuroscience area with the other fields described previously, the contributions are still less common [3].

In terms of analysis of brain activity, DA has been mainly used for three purposes: state estimation, modeling, and noise reduction. In the first one, searching for the relationship between brain activity and a task of interest is the main purpose. For example, Ref. [8] estimates brain connectivity in different regions to improve source localization techniques. In [9], the correlation between cortical dynamics and seizures was analyzed to gain a better understanding of epilepsy. In terms of using DA for data modeling, most of the work is related to signal improvement for brain-computer interfaces (BCI) [10,11], and to study the brain during certain conditions like sleeping [12]. Finally, for the goal of eliminating noise in brain data, some examples of how DA has been used can be found in [13,14,15].

For the analysis of the brain signal, the quantitative electroencephalogram (QEEG) features can be used direct- or indirectly with the DA methods. Direct usage refers to using a main characteristic of the input data itself, such as its amplitude (the most used in the studies described). The indirect approach uses modeling parameters obtained from the data that represent the task of concern, for example, the QEEG coherence (which is part of our research proposal). Another indirect form is to use the results obtained from the DA method and calculate other variables. This allows the obtaining of additional information of the brain signals, e.g., the power spectrum (also used in the majority of the studies). Here we consider that with the use of two QEEG characteristics, along with DA, we can obtain more information about the brain areas involved in the task of interest, as well as analyze changes in the behavior of different brain rhythms related to the same task. With this, the experts of the field can make a better interpretation of the analysis outcome regarding the event of concern.

In this paper, we propose a methodology that uses DA with the EnKF method along with the power spectrum and QEEG coherence as features of interest. Although the power of different frequency bands is used for evaluating a significant change in the phenomenon of interest, the coherence is used for selecting the most relevant sensors for each user related to this event. With this information, we can better understand the behavior of the brain rhythms in the different brain areas related the task of interest. As described previously, this approach can be helpful to the experts of the area of neurosciences, like neurologists and academics, to make diagnosis and medical decisions related to patient performance. In this research, we focus our attention in showing the applicability of our proposed technique through the analysis of cognitive abilities that have been previously measured in two different datasets previously analyzed in [16,17]. Both datasets acquire QEEG data related to the development of a cognitive activity. Hence, this paper is organized as follows: In Section 2, we explained the methodology for the analysis of the brain signals. We include the description of the datasets used, the selection of the relevant sensors with the coherence feature, the use of EnKF as our DA method, and the statistical analysis performed with the Wilcoxon signed-rank test. The results obtained are presented in Section 3; in Section 4, the results are discussed; and concluding remarks are provided in Section 5.

## 2. Materials and Methods

**Figure 1:**
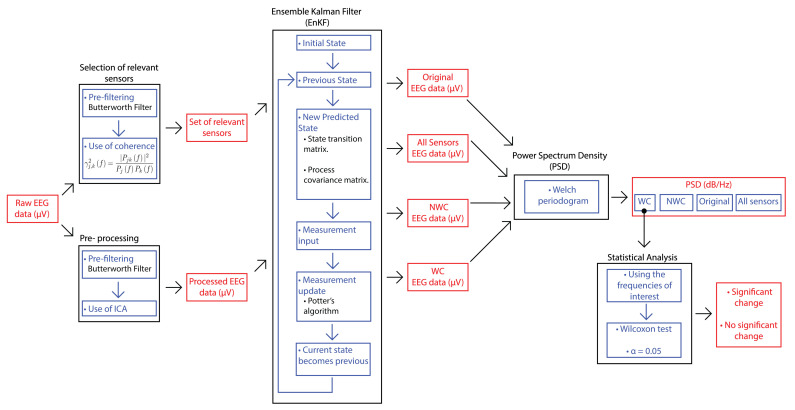
The proposed methodology that combines EnKF and QEEG features.

An overview of the methodology proposed in this paper is shown in Figure 1. The first step is based on the coherence-based method proposed in [18], in which the EEG data was used for selecting sensors that provide the most relevant information for the brain task of interest. Since the coherence expresses the correlation between two signals in the frequency domain, it gives an idea of the interconnection level that two different brain areas had. Moreover, the signal is filtered with a bandpass and independent component analysis (ICA) for eliminating artifacts. With the relevant sensors and the filtered signal, the EnKF is applied. For this, a Python program was developed with different libraries like Numpy. The main reason for using this programming language along with these libraries is two-fold: the reduction of computer memory for storage and the easier manipulation of matrix structures since Numpy is implemented in C. Hence, matrix operations are faster to compute [19]. Our Python implementation is freely available in [20]. The program returns the original signal and the output of applying EnKF in three scenarios: considering all the electrodes, considering the relevant ones (referred to as winner channels or WC), and not considering these electrodes (not winner channels or simply NWC). Then, the power spectrum density (PSD) is obtained from these outcomes. Finally, the Wilcoxon signed-rank test is computed with the PSD of the relevant sensors to find out if there was a significant change in the frequency range selected regarding the phenomenon of interest.

A more detailed description of the main components of our proposed method is provided in the following subsections. Specifically, more details on the EEG data we used are given, as well as an explanation on the use of coherence for selecting the relevant electrodes for each user, the use of EnKF with EEG recordings, and the statistical analysis of the results.

### 2.1. EEG Data

To demonstrate the viability of the proposed methodology, real EEG data were used from two different datasets. In both, the activities performed were related to cognitive skills. For future references, we will refer the dataset from [16] as LGR and the one from [17] as DM (related to the authors’ names of each dataset). In Table 1, different characteristics of each dataset are described, such as the headset used for acquisition, its sampling rate, and the number of recordings. Regarding the users in Dataset LGR, we used the first five participants. The reason is that they have the same number of sessions recorded and consistency in performing the task, compared to the rest of the users.

LGR was acquired at Universidad de las Américas Puebla (UDLAP) as part of the PhD thesis research of the first author. The five participants used for this study are undergraduate students (average 19.6 years ± 1.74) from the same university. The users were asked to focus on moving a 3D figure and imagine that they were pushing the object away for five seconds. When they finished the activity successfully, they received an auditory stimulus. Each session consisted of three brain recordings: before, during, and after the activity. As presented in Table 1, the headset used was the Emotiv EPOC+, with fourteen sensors and two references (AF3, F7, F3, FC5, T7, P7, O1, O2, P8, T8, FC6, F4, F8, AF4, CMS, and DRL). The five users performed this task in 19 sessions, depending on their schedule and time availability. The main goal was to analyze the possible change in the PSD of the individual alpha peak frequency (IAPF), the frequency in the range of alpha with the largest value in the spectrum [21], by performing a concentration task. This improvement in the spectrum has positive effects on cognition and memory enhancement, as well as clinical treatment [22,23].

DM was acquired at the Center for Research and Advanced Studies (Cinvestav) Monterrey’s Unit, as part of a MSc thesis research. We selected this dataset to analyze a cognitive task combined with motor movement. Another reason was related to study other frequency bands besides the alpha rhythm. The ten users from the dataset are part of the same institution (average 29.3 years ± 5.7). The participants performed 12 different lessons for learning to type in a computer with the Colemak keyboard layout [24]. The difficulty of the task increased as the lessons progressed. The users repeated each of them five times, and in lesson four, eight, and eleven, their brain signals were recorded. As described in Table 1, the headset used was the B-Alert X10, with nine sensors (F3, Fz, F4, C3, Cz, C4, P3, POz, and P4). The main aim was to study the spectrum behavior in different frequency bands during the process of learning a new skill. As a result, they reported a significant decrease in the PSD of beta and gamma bands. This change in the former rhythm is related to motor learning activities, and the decrease in the latter band can be associated with temporal binding, which is the ability to group separate events that occur at different lapses of time [25]. Both datasets are freely available at [26,27].

For this research, we considered the recordings before and after the activity from all sessions in Dataset LGR. Likewise, we used the first and last repetition from the lessons recorded (4, 8, and 11) in Dataset DM. Such selection was made as we are interested in finding if there was a significant change in the user’s PSD as a result of performing an activity related to the corresponding cognitive ability (concentration and learning a new skill, respectively). For future references, the EEG recordings from Dataset LGR will be referred to as pre- and post-recording, while the EEG registrations from Dataset DM will be addressed as the #1- and #5-recording. With these recordings, we can perform the selection of most relevant electrodes to the task of concern.

### 2.2. Selection of Relevant Sensors

In this work, we consider as *relevant sensors* those that are more likely to have a significant coherence value in the selected frequency range related to the phenomenon of interest. To find these electrodes, the following methodology was used:

- Data was filtered with a bandpass Butterworth filter of fourth order, with cutoff frequencies of 1 and 63 Hz.
- The coherence-based electrode selection method described in [18] was adapted to be applied to the datasets used in this work, considering the characteristics of the cognitive activity performed. This methodology takes into account the connectivity between different brain areas by analyzing the values of coherence between different electrodes in the frequency band of interest. In the case of Dataset LGR, the brain rhythm analyzed was the alpha band (8–12 Hz), while in Dataset DM it was beta (13–29 Hz) and gamma (30–40 Hz) bands. For each frequency, the coherence was evaluated as follows:
  – From the *m* available electrodes, all possible combinations of three are assessed with the binomial coefficient $\left(C_{3}^{m}\right)$,
  – For each subset of three electrodes, the coherence value was computed between all pairs of electrodes, generating three coherence values: $\gamma_{1,2}\left(f\right)$, $\gamma_{1,3}\left(f\right)$, $\gamma_{2,3}\left(f\right)$, which are computed as
    (1) $$\gamma_{j,k}^{2}\left(f\right)=\frac{|P_{j,k}{\left(f\right)|}^{2}}{P_{j}\left(f\right)P_{k}\left(f\right)},$$
    where *f* is the frequency, *j* and *k* are the sensors to analyze, $P_{j,k}\left(f\right)$ is the cross-spectral density (CSD), $P_{j}\left(f\right)$ and $P_{k}\left(f\right)$ are the PSD (i.e., the auto-spectral densities, or ASD) of *j* and *k*, respectively. The ASD of the signal recorded with one electrode is given by:
    (2) $$P_{\left\{*\right\}}\left(f\right)=\sum_{\tau =-\infty }^{\infty }E\left\{x_{\left\{*\right\}}\left(n\right)x_{\left\{*\right\}}\left(n-\tau \right)\right\}e^{-j2\pi \tau f},$$
    where $\left\{*\right\}$ indicates either *j* or *k*, $E\left\{\cdot \right\}$ is the expected value, and n=1,2,…,N time samples. Similarly, CSD is given by:
    (3) $$P_{j,k}\left(f\right)=\sum_{\tau =-\infty }^{\infty }E\left\{x_{j}\left(n\right)x_{k}\left(n-\tau \right)\right\}e^{-j2\pi \tau f}.$$
  – To assure that each one of the obtained values expressed a true connectivity, the significance of those coherence values was evaluated according to [28]. Here, pairs of surrogate time series are generated as realizations of two linearly independent stochastic processes. Those surrogate series share features of the original EEG recordings but are uncoupled. Then, the coherence between each pair and its frequency histogram are calculated. Finally, the threshold is set at the 95-percentile of the sampling distribution. If the actual signals from the subset of sensors being tested had a significant coherence (i.e., $\gamma_{j,k}^{2}\left(f\right)>$ threshold) in all three values, the subset was stored.
  – This process was repeated for all trials of the EEG recordings.
- Since the coherence is a frequency-based metric, the subsets were evaluated in the selected frequency range (Dataset LGR: 8–12 Hz; Dataset DM: 13–29 Hz and 30–40 Hz). The subset that had 100% of appearance in all the frequency range in the EEG recordings, was taken into account as a possible candidate,
- The subset that had more repetitions in all the studies was selected as the relevant one.

With the relevant set of electrodes for each user, we performed DA by using the EnKF and the brain recordings.

### 2.3. Data Assimilation with EnKF

The EnKF is a Monte Carlo-based implementation of the KF for high-dimensional and nonlinear state estimation problems [3,29]. Compared to the main KF (described in Appendix A), the EnKF increases the numerical precision of the KF, and the computational requirements are affordable since it does not need derivatives for calculating the state and observation matrices like in other variants like EKF [30,31,32].

Here, the state $x_{t}^{p}$ and the observation $y_{t}$ are calculated according to Equations (A1) and (A2). The state transition matrix *F* is calculated with Taylor series [33], and the transformation matrix *H* is modeled based on the relevant sensors of each user. Compared to the traditional KF, EnKF transforms the process covariance matrix $P_{t}$ into squared root matrices (SRM) as follows:

(4) $$P_{t}=S_{t}S_{t}^{T},$$

where $S_{t}$ is a SRM with size *m* × *m*. By applying this transformation, we reduce the computational burden in a real-time system implementation with greater precision, and it solves the problem of the round-off error’s sensitivity [31]. Please note that in the following steps, we use $S_{t}$ instead of $P_{t}$. To calculate $S_{t}$, we can use the Cholesky factorization algorithm or the $LDL^{T}$ decomposition [31,34]. For this work, we used the $LDL^{T}$ decomposition since we avoid the use of square-root operations for the diagonal elements [35].

For calculating the new predicted $S_{t}^{p}$, hence $P_{t}^{p}$ (Equation (A3)), it is important to perform the next calculation:

(5) $$[\begin{array}{c} {\left(S_{t}^{p}\right)}^{T}\\ 0 \end{array}]=B* [\begin{array}{c} {\left(S_{t-1}\right)}^{T}F^{T}\\ Q_{t-1}^{T/2} \end{array}],$$

where *B* is a 2m×2m orthogonal matrix that can be computed through different methods such as Householder, Gram-Schmidt, modified Gram-Schmidt, and Givens rotation [31]. In this work, we used the Givens rotation, since it is less sensitive to scaling problems, and it is more flexible for zero-converting [36,37]. With this method, *B* is defined as follows:

(6) $$B= [\begin{array}{cc} c & s\\ -s & c \end{array}],$$

where *c* and *s* indicate the cosine and sine functions of an angle, respectively. According to [36], the trigonometric functions are not required since its application is as follows:

(7) $$[\begin{array}{cc} c & s\\ -s & c \end{array}] [\begin{array}{c} a\\ b \end{array}]= [\begin{array}{c} r\\ 0 \end{array}],$$

where $r=\sqrt{a^{2}+b^{2}}$, c=a/r and s=−b/r. The complete process can be found in Appendix B as Algorithm A1.

Another important component of the EnKF is the update of the process covariance matrix $P_{t}$ and state $x_{t}$ (A5,A6), along with the calculation of the Kalman gain in (A4). Hence, James E. Potter’s algorithm (used for NASA’s Apollo space program) was implemented but modified for vectorial measurements [31]. According to [38], this method guarantees the positivity of the computed error covariance. The process is shown in Appendix C as Algorithm A2. The outcomes of this method become the previous values and the process is repeated. To perform DA of our data through EnKF, the following methodology was applied:

- As a preprocessing step, Dataset LGR was filtered with a bandpass Butterworth filter of 4th order in the range [1,63] Hz while for Dataset DM, a bandpass Butterworth filter of 5th order was used in the range [1,100] Hz. These cutoff ranges were selected according to the $F_{s}$ of each dataset. For both datasets, independent component analysis (ICA) was performed to remove movement and sensor artifacts. Please note that regardless of this filtering process, measurement noise $z_{t}$ was taken into account to calculate the sensor noise covariance matrix *R* used in Potter’s algorithm.
- Then, the EnKF was applied in the signal and we analyzed three cases for comparison purposes: (i) using measurements from all sensors, (ii) considering only those signals from relevant electrodes (according to the process in Section 2.2, and (iii) leaving out relevant sensors. The main reason to contemplate these situations is to see the spectral contribution from different brain regions, as well as assessing the advantage of sensor selection given that as described previously, these electrodes are significantly correlated with the frequency bands associated with the task of interest. Under those conditions, PSD was obtained for those cases through Welch’s method with a 50% overlap.

Plotting the PSD of different sessions allow us to have an illustrative representation of the results to compare them. The main goal was to analyze, depending on the brain rhythm of interest, a possible increase or decrease in the PSD of each user. Nevertheless, it was necessary to evaluate if this significant change was presented because of the activities performed in each study and not by chance. We did this evaluation with the Wilcoxon signed-rank test.

### 2.4. Statistical Analysis

Once we performed the DA method, we evaluated whether there was a significant change in the PSD values due to the performance of cognitive tasks. For this work, we used the Wilcoxon signed-rank test for two reasons: the PSD data does not have a normal distribution and we wanted to examine paired groups [39,40]. For Dataset LGR, pre- and post-recordings were compared, while for Dataset DM, we were interested to find differences between trial #1 and #5 in each of the three sessions measured. Please note that for this statistical analysis, we used only the signal that considers the relevant sensors. The reason was to know how these electrodes contributed or not with the user’s performance regarding the PSD, since these are the most correlated with the task of concern.

All statistical tests were carried out with a 5% significance level. We were particularly interested in looking for significant differences in the PSD values at alpha, low- and high-beta, or gamma bands, given the previously reported findings in [16,17], respectively. Please note that a change in the frequency band was considered to be relevant when the majority of its PSD values (with 1 Hz resolution) turned out to have significant differences between the groups.

## 3. Results

In this Section, we present the results of the proposed analysis. For each dataset, we display the outcomes corresponding to the channel selection, next the PSD resulting from the EnKF in the three scenarios described previously, and at last, the statistical analysis.

### 3.1. Dataset LGR

Please note that for this dataset, the headset used was the Emotiv EPOC+ for the brain recording. With the 14 sensors available, we have 364 possible combinations of three electrodes. These subsets were evaluated with the methodology in Section 2.2, and the one that had more repetitions in all the EEG recordings was selected as the relevant one. Table 2 displays the position of the relevant sensors for each user.

As it can be seen, at least one relevant electrode was located on the frontal area for all users. This is an expected result since the alpha band, specifically the IAPF, was already been identified in the frontal lobe in relationship to cognitive activity [41]. In the case of Users 4 and 5, they presented more than three relevant sensors because they had a tie of different subsets that got repeated the most number of times in all sessions. For instance, User 5 had a tie between subset *FC5, F8, AF4*, and *T8, F4, AF4*. Also note that all users had *AF4* as relevant. According to [42], the right dorsolateral prefrontal cortex (DLPFC) is related to the cognitive processes by allowing people to control their actions, and its activity is known to increase during working memory tasks as a reflection of focus and attention [43,44].

**Figure 2:**
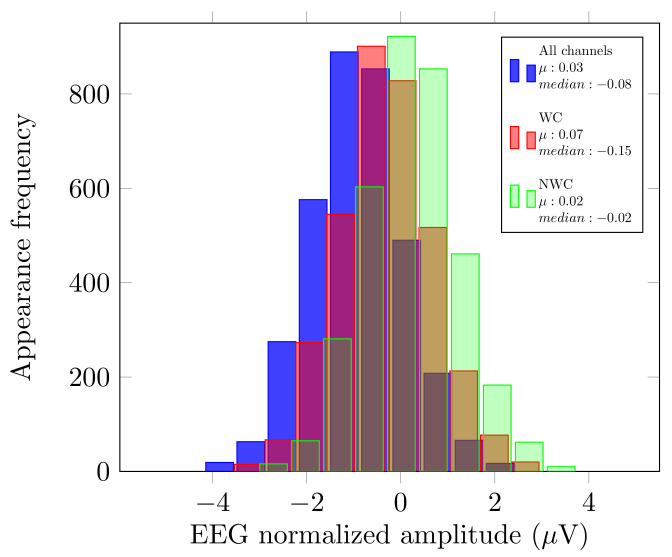
Histograms of User 1 pre-recording.

As described in Section 2.3, we analyzed the original signal, and the use of EnKF in three different cases: all the sensors, only the relevant sensors, and without these electrodes. Since these outcomes were calculated with distinct numbers of sensors (m=14,3,11), we compared the mean (µ) and median from these measurements to verify that no bias had been introduced by possible outliers. Figure 2 displays an example of this comparison for User 1.

As displayed in Figure 2, the µ and median of each of the histograms have very close values. With these variables, we can see that there was no bias if we considered fewer or more electrodes in our EEG analysis.

**Figure 3:**
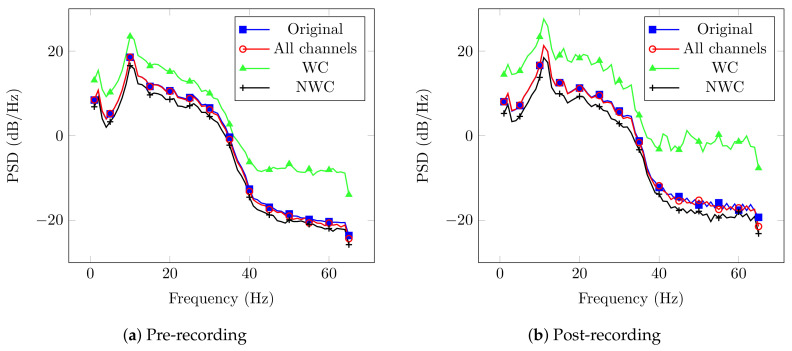
PSD results from EnKF outputs of User 1.

Afterwards, every spectrum from the original signal and those after the proposed DA technique were computed. An example of this process is shown in Figure 3 for User 1, which contains four curves that represent the original signal, all sensors, only relevant sensors, and not considering them.

Please note that the PSD for the WC case has higher magnitude in comparison to the other cases. This behavior is supported by the fact that its result is more correlated with the activity of interest. In contrast, the NWC yielded lower power, since it does not contain the relevant electrodes. Another thing to remark is the behavior of the original signal and the case that considers all sensors. Both curves have the same performance, and we expected this behavior since the EnKF is modeling and predicting the pre-filtered EEG recording.

**Figure 4:**
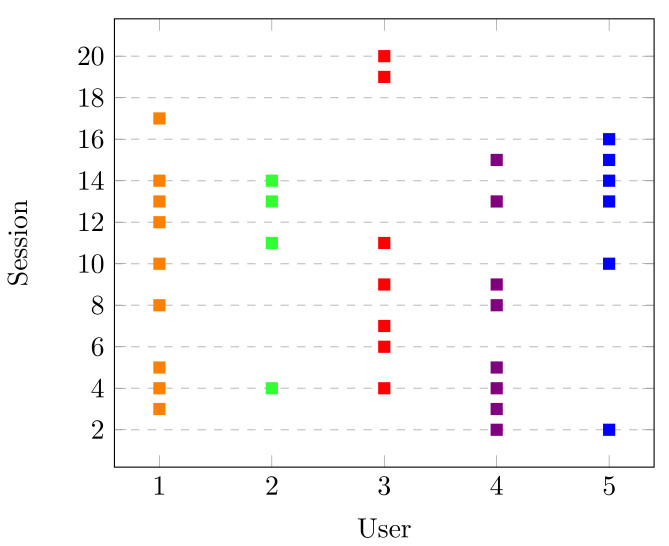
Sessions with significant change in Dataset LGR.

As described in Section 2.4, the statistical testing was performed to find significant changes for all users and sessions. Please note that the main goal of the Dataset was to study a possible increase in the IAPF’s spectrum with the concentration task. The statistical results can be seen in Figure 4, where the *x*-axis represents the users and the *y*-axis is each of the sessions recorded. There, the points represent in which sessions the participants had a significant increase in the alpha band. We note that all users had this performance in more than one session. This is an expected result since the alpha band is related to cognitive processes like concentration [41]. Moreover, we can appreciate that in consecutive sessions the participants had the significant increase, and then there is a range of sessions without a change. For example, User 1 had sessions 3, 4, and 5 highlighted, and she did not have another significant increase until session 8. As well, the participants have less than half of the sessions performed with a significant change. This behavior is discussed in detail in Section 4.

### 3.2. Dataset DM

For this dataset, the authors used the B-Alert X10 headset for the EEG recordings. In this case, with the nine available sensors, we had 84 possible combinations of three electrodes. With the #1 and #5 recordings and the procedure described in Section 2.2, the relevant sensors were obtained and they are displayed in Table 3. Likewise, Dataset LGR, some of the users had more than three significant electrodes. For example, Users 4 and 9 had a tie between subset *C3, P3, POz*, and *Cz, POz, and F4*. As described in Section 3.1 the reason of these outcomes is that they had a tie of different subsets that repeated the most in the study. Also note that nine of ten participants had in common subset *C3, P3, POz*. Such set of sensors cover and acquire the activity of the primary motor cortex, whose activation is known to be implicated in the process of skilled movement [45].

**Figure 5:**
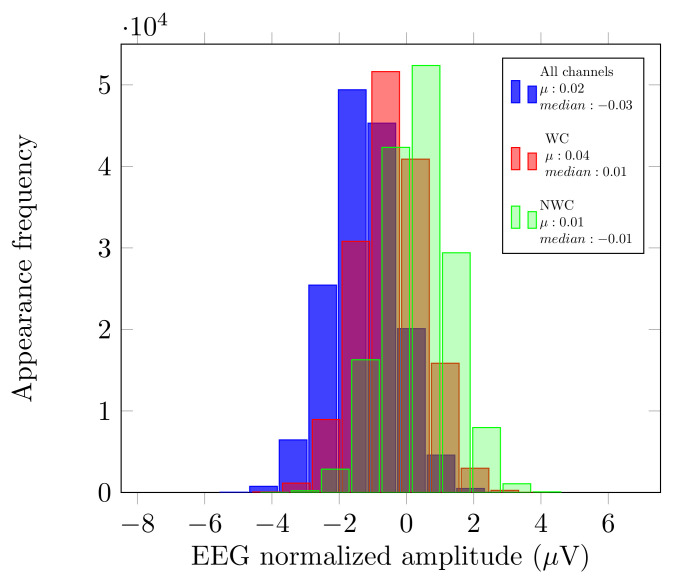
Histograms of User 5 first recording.

Just like Dataset LGR, we analyzed the original signal, and the use of EnKF in the three scenarios described previously. Please note that since the authors used the B-Alert System, the number of sensors differs (m=9,3,6). We also compared the mean (µ) and median from the EnKF measurements. The aim was to verify that no bias had been introduced by possible outliers. Figure 5 displays this comparison for User 5 and, since the µ and median have similar values, we can say that there was no bias if we considered fewer or more electrodes.

**Figure 6:**
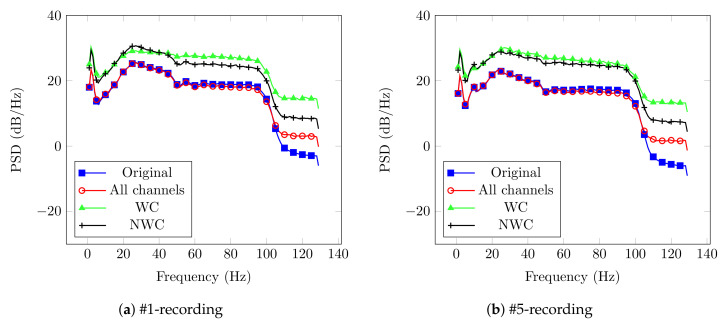
PSD results from EnKF outputs of User 5.

An example of the results obtained with the EnKF are shown in Figure 6 for the case of User 5. In the same fashion as with Dataset LGR, the figure contains four cases: the original signal, all sensors, WC, and NWC.

Again, the plot corresponding to WC stands out as the one with higher power for most of the frequencies; however, NWC curve is on top for some frequencies and not far away of WC. Such behavior could be related to different factors that affect the user’s performance and, in consequence, the power of certain frequency bands [46]. Nevertheless, this behavior could be related to the selection of the relevant sensors. As described in Section 2.2, the subsets needed to have a 100% appearance in all the frequency range in the brain recording so we could consider it as a candidate.

**Figure 7:**
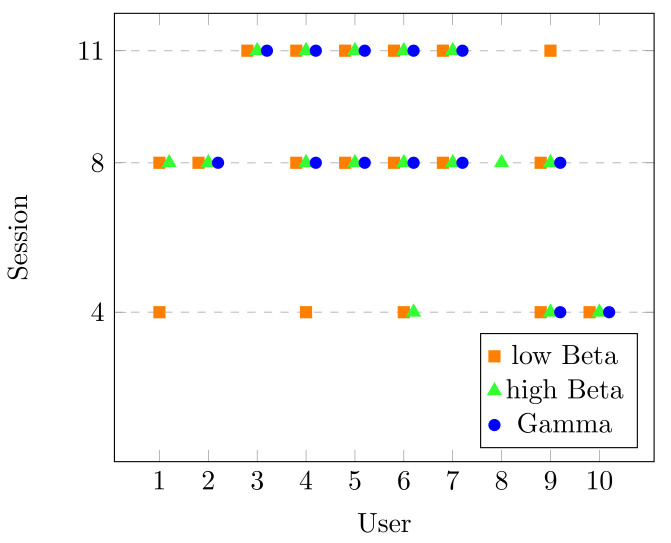
Sessions with significant change in Dataset DM.

Please note that the main goal of this dataset was to study the spectrum behavior in different frequency bands during the process of learning a new skill. They reported a significant decrease in the PSD of beta (13–29 Hz) and gamma (30–40 Hz) bands. With this information, the corresponding statistical analysis for beta (low and high) and gamma bands was performed on selected channels and significant changes found are presented in Figure 7. Like in Figure 4, the *x*-axis presents the users of the dataset and the *y*-axis is every lesson recorded. Moreover, each point (according to each frequency band) displays where the users had the desirable performance. Please note that most of the users show significant changes in the beta band (either low, high, or both) in most of the lessons. This agrees with beta band being related to motor learning activities, as in the case of the typing task. The gamma band also had a significant change, which could be associated with temporal binding, which is the ability to group separate events occurring at different lapses of time [25].

## 4. Discussion

Our proposed analysis allowed us to obtain new insight regarding the cognitive processes taking place in each of the datasets.

Regarding the selection of relevant sensors, the process could identify electrodes with a significant coherence value in the frequency bands related to the tasks of interest. Please note that the majority of the participants had sensors whose location over the head cover brain areas associated with the concentration and the process of learning a new skill. For DM, beta and gamma bands in the primary motor cortex (central and parietal lobes) are related to the acquisition and performance of the skilled movement. The activation of these areas can be seen in the subset of sensors *C3, P3, POz*, which appeared in most of the users. Nevertheless, three participants (3, 4, and 9) had at least one frontal electrode. According to [47], beta rhythm in the frontal lobe is associated with cognitive tasks regarding decision making, which could be presented during the typing activity. Furthermore, gamma rhythm in the same brain area is correlated with movement execution [48], and phonological processing [49].

For the EnKF outcomes, the graphs displayed contain four different curves showing the original signal, the use of all electrodes, and considering or not the relevant sensors. The main result there is that magnitude of the PSD when WC are considered is greater than the one for other channel’s combinations. This behavior is the result of the use of electrodes mainly correlated with the corresponding frequency bands studied.

In regards to the statistical results, the users in LGR had less than half of the sessions with a significant increase in the alpha band. This outcome could be the result of two different aspects: overtraining and cognitive demand. Overtraining could be linked to an excess of practice, which leads to a decrease in the user’s performance. This performance could have a possible increase in the progression of the experiment [50,51]. This behavior can be seen, for example, in Users 3, 4, and 5 in Figure 4. Cognitive demand might be related to the memory demand and attentiveness that increases power in alpha band [50,52]. Some users in Figure 4 did not show a significant increase over the last sessions, and this is possibly related to the low cognitive demand because of the activity repetitiveness, as the concentration activity was the same in all the sessions. In Dataset DM, we can see that all users had a significant change in beta (low, high, or both) in most of the sessions. This behavior is in agreement with the activation of the primary motor cortex (due to typing) and the parietal activation, where a variety of cognitive tasks like working memory and attention converge [53]. Regarding the changes in the gamma band, this can be certainly related to the process of looking at a paragraph, remembering it, and then writing it with a specific keyboard layout. Besides this, the gamma band is known to control coupling perception, which is associated with motor skills and learning [54,55].

## 5. Conclusions

In this paper, we proposed an analysis process of brain activity signals through DA foundations, specifically those related to the EnKF, and QEEG features. We showed the applicability of the proposed method through a series of numerical examples in real EEG data, corresponding to cognitive processes. The results show the advantage of using the proposed method together with channel selection techniques in order to provide the most relevant information from data; hence, those most likely related to the cognitive process of interest. In both databases here analyzed, we were able to confirm the observations previously made on the relationship of changes in different brain rhythms as the cognitive process progressed.

Future work will include a validation of the proposed methodology with the assistance of neuroscience experts (e.g., neurologists and academics). For this, we need to perform more tests with other brain signals related to different human skills. Moreover, we contemplate the creation of a user interface so the outcomes can be interpreted easily by the experts. With these improvements, the methodology can be used for evaluating the progress of the patients regarding cognitive ability, motor skills, neurological disorders, among others. 

## Figures and Tables

**Table 1:** Features of the datasets analyzed with the proposed methodology.

	LGR	DM
Number of subjects	5	10
Headset	Emotiv EPOC+	B-Alert X10
Number of sensors	14	9
Sampling rate (Hz)	128	256
Sessions recorded	19	3
Recordings per session	3	5
Frequency bands (Hz)	Alpha (8–12)	Beta (13–29) and gamma (30–40)

**Table 2:** Relevant electrodes for each participant in Dataset LGR.

User	Relevant Sensors
1	F4, F8, AF4
2	F3, FC6, AF4
3	FC5, F4, AF4
4	F3, FC5, F4, F8, AF4
5	FC5, T8, F4, F8, AF4

**Table 3:** Relevant electrodes for each participant in Dataset DM.

User	Relevant Sensors
1	C3, P3, POz
2	C3, Cz, P3, POz
3	F3, C3, Cz
4	F4, C3, Cz, P3, POz
5	C3, Cz, P3, POz
6	C3, P3, POz
7	C3, P3, POz
8	C3, P3, POz
9	F4, C3, Cz, P3, POz
10	C3, P3, POz

## Abbreviations

The following abbreviations are used in this manuscript:

ASD	Auto-spectral density
BCI	Brain-computer interface
Cinvestav	Center for Research and Advanced Studies
CSD	Cross-spectral density
DA	Data assimilation
DLPFC	Dorsolateral prefrontal cortex
EEG	Electroencephalogram
WC	Winner channels
EKF	Extended Kalman filter
EnKF	Ensemble Kalman filter
IAPF	Individual alpha peak frequency
ICA	Independent component analysis
KF	Kalman filter
NWC	Not winner channels
PSD	Power spectrum density
QEEG	Quantitative electroencephalogram
SRM	Squared root matrix
UKF	Unscented Kalman filter
UDLAP	Universidad de las Américas Puebla
WC	Winner channels

## Appendix A. Kalman Filter (KF)

Kalman filter (KF), also known as linear quadratic estimator, is a recursive method whose principal goal is to estimate the state of a phenomenon of interest when the measured data can contain uncertainty errors [56]. It estimates the state $x_{t}^{p}\in R^{m}$ of a discrete-time controlled process, where *p* denotes a predictor, *t* is the time step and *m* is the number of elements. It is driven by the following linear stochastic calculation [57]:

(A1) $$x_{t}^{p}=Fx_{t-1}+w_{t},$$

with a measurement $y_{t}\in R^{n}$, where *n* is the number of elements to study. This variable is given by,

(A2) $$y_{t}=Hx_{t}+z_{t},$$

where *F* is a state transition matrix with size *m* × *m*. According to [58,59], *F* can be achieved through Taylor series, Padé series, inverse Laplace transform, matrix decomposition methods, among others. *H* is a transformation matrix with size *n* × *m*, $w_{t}$, and $z_{t}$ are the process and measurement noise, respectively. We consider the former as the noise that is originated from the input data itself, while the latter is the noise that is generated in the sensors that read the data. Both are zero-mean Gaussian processes and column vectors with size *m* × 1 and *n* × 1, respectively. This filter operation can be summarized in five steps:

- An initial state is given to the filter, which includes the state ($x_{0}$) and the process covariance matrix ($P_{0}$);
- Then, a new predicted state and process covariance matrix are calculated, the former using (A1) and the latter by:
  (A3) $$P_{t}^{p}=FP_{t-1}F^{T}+Q_{t},$$
  where $Q_{t}$ is the process noise covariance matrix with size *m* × *m*, and it is determined with $w_{t}$;
- The measured input is considered and Kalman gain is calculated. The former is given by (A2) and the latter is obtained through the following:
  (A4) $$K=\frac{P_{t}^{p}H^{T}}{HP_{t}^{p}H^{T}+R},$$
  where *R* is the sensor noise covariance diagonal matrix with size *n* × *n*, and it is determined with $z_{t}$;
- The process state and covariance matrix are updated as follows:
  (A5) $$P_{t}=\left(I-KH\right)P_{t}^{p},$$
  (A6) $$x_{t}=x_{t}^{p}+K\left(y_{t}-Hx_{t}^{p}\right),$$
  where *I* is the identity matrix with size *m* × *m*;
- The results of (A5) and (A6) become the previous values. Next, the process is repeated starting from the second step.

## Appendix B. Givens Rotation

**Algorithm A1** Givens rotation
1: **Input:** $F,Q_{t-1},S_{t-1}$ 2: **Output:** $S_{t}^{p}$ 3: m← size of $S_{t-1}$ 5: $U\leftarrow [\begin{array}{c} F^{T}•S_{t-1}^{T}\\ Q_{t-1}^{T/2} \end{array}]$ ▹ size 2m×2m 7: **for**j←1 to *m* **do** 8: **for** i←2m:−1:j+1 **do** 9: B←I ▹ size 2m×2m 10: $a\leftarrow U_{i-1,j}$ 11: $b\leftarrow U_{i,j}$ 12: **if** b=0 **then** 13: c←1 14: s←0 15: **else** 16: **if** abs(b)>abs(a) **then** 17: r←a/b 18: $s\leftarrow 1/\sqrt{(}1+r^{2})$ 19: c←s×r 20: **else** 21: r←b/a 22: $c\leftarrow 1/\sqrt{(}1+r^{2})$ 23: s←c×r 24: **end if** 25: **end if** 26: $B_{\left[i-1,i],[i-1,i\right]}= [\begin{array}{cc} c & -s\\ s & c \end{array}]$ 27: $U\leftarrow B^{T}•U$ 28: **end for** 29: **end for** 30: $S_{t}^{p}\leftarrow U_{\left[1:m],[1:m\right]}$

## Appendix C. Potter’s Algorithm

**Algorithm 2** Potter’s algorithm.
1: **Input:**$x_{t}^{p}$, $S_{t}^{p}$, $y_{t}$, *H*, *R* 2: **Output:**$x_{t}$, $S_{t}$ 4: **Initialize**$x_{0,t}\leftarrow$$x_{t}^{p}$ and $S_{0,t}\leftarrow$ $S_{t}^{p}$ 6: **for**i←1 to *n***do** ▹*n*: Size of $y_{t}$ 7: $H_{i}\leftarrow$*i*th row of *H* 8: $y_{i,t}\leftarrow$ *i*th element of $y_{t}$ 9: $R_{i}\leftarrow$ variance of *i*th measurement 10: $\phi_{i}\leftarrow$ $S_{i-1,t}^{T}H_{i}^{T}$ 11: $a_{i}\leftarrow$ $1/(\phi_{i}^{T}\phi_{i}+R_{i})$ 12: $\gamma_{i}\leftarrow$ $a_{i}/\left(1\pm \sqrt{a_{i}R_{i}}\right)$ 13: $S_{i,t}\leftarrow$ $S_{i-1,t}\left(I-a_{i}\gamma_{i}\phi_{i}\phi_{i}^{T}\right)$ 14: $K_{i,t}\leftarrow$ $S_{i,t}\phi_{i}$ ▹ Kalman gain of *i*th measurement 15: $x_{i,t}\leftarrow$ $x_{i-1,t}+K_{i,t}\left(y_{i,t}-H_{i}x_{i-1,t}\right)$ 16: **end for** 18: **Set**$x_{t}\leftarrow$$x_{m,t}$, $S_{t}\leftarrow$$S_{m,t}$
